# A quantitative analysis of trauma patients having undergone plastic surgery

**DOI:** 10.1371/journal.pone.0272054

**Published:** 2022-08-15

**Authors:** Nam Kyu Lim, Jae Hee Yoon

**Affiliations:** 1 Department of Plastic and Reconstructive Surgery, Dankook University College of Medicine, Cheonansi, Chungcheongnamdo, Republic of Korea; 2 Department of Plastic and Reconstructive Surgery, Dankook University Hospital, Cheonansi, Chungcheongnamdo, Republic of Korea; UTMB: The University of Texas Medical Branch at Galveston, UNITED STATES

## Abstract

**Purpose:**

While plastic surgeons have been historically indispensable in reconstruction of posttraumatic defects, their role in Level I trauma centers around the world has not yet been clearly approved. This study aims to assess the contribution of plastic surgeons in major trauma care by evaluating the characteristics of trauma patients underwent plastic surgery at a Level I trauma center.

**Method:**

From November 2014 to October 2020, we conducted a retrospective review of our hospital’s Trauma Registry System for patients with an Injury Severity Score (ISS) of 9 or higher. Of all of 7174 patients, the plastic surgery (PS) department treated 870 patients; the 6304 patients not treated by the PS were classified as the Non-PS. Then, we performed propensity score matching to reduce the statistical bias, after the death in the emergency room and the missing value were considered exclusion criteria.

**Result:**

The mean ISS showed no significant difference between two groups (16.29 ± 7.04 in the PS vs. 16.68 ± 9.16 in the Non-PS, *p* = 0.3221). According to investigate the Abbreviated Injury Scale, both head and neck (65.0%) and face (46.4%) categories showed significantly higher in the PS group than the Non-PS group (*p* < 0.0001), and its contribution ratio was 2.151 and 21.822 times, respectively.

**Conclusion:**

This study revealed the specialty of plastic surgery was face area in trauma care. We thus argue that plastic surgical care is imperative for trauma patients, and expect to be implicated in trauma system planning.

## Introduction

Armed forces engaging in battlefields of the early 20th century faced unprecedented dangers due to advances in weaponry and tactics. Added to the escalated risks, in trench warfare, an exposed face immediately became a target, resulting in a surge in the incidence of facial injuries. Such were the circumstances when the English-trained surgeon Sir Harold Gillies, known as “the Father of Modern Plastic Surgery,” set up Queen’s Hospital in Sidcup, the first hospital dedicated to treating facial injuries. In the next several years, the hospital conducted over 11,000 surgeries on soldiers with maxillofacial trauma [1,2]. Nevertheless, even with substantial advancement of plastic surgery after the end of World War I, in the 1920s the profession was still yet to be clearly defined as a separate discipline. It was only in the late 1930s that the American Board of Plastic Surgery (ABPS; the predecessor of the American Society of Plastic Surgery) took form and later began administering exams to qualify plastic surgeons [3]. Its counterpart in the United Kingdom (UK), the British Association of Plastic Reconstructive and Aesthetic Surgeons (BAPRAS), was established even later in 1944 with help from Sir Harold Gillies [4]. As such, plastic surgery has been recognized as a specialty in its own right less than a century ago.

However, thanks to much academic and clinical endeavor, plastic surgeons today play an integral role over a diversity of fields, from reconstructive to aesthetic surgery. Plastic surgeons are equipped with the skill sets required to address traumatic injuries, including management of soft-tissue injury, fractures in facial structures, neurovascular injury, and salvaging extremities [5–8]. Developments in diagnostic and therapeutic devices over the past 50 years, such as the evolution of imaging, microscope, dressing materials, and surgical instruments are also conducive to plastic surgeons’ role in treating trauma patients [9].

One of the world’s leading causes of death is trauma, particularly for individuals of reproductive age. Such deaths translate to an economic burden on society, considering the lost prime working years, among other costs; therefore, much government funding has been spent on reducing both morbidity and mortality of trauma patients [7,10]. Approximately half of the 12 million traumatic wounds treated in emergency rooms each year in the United States (US) involve injuries to the head and neck [11–13]. Head injuries, a serious health concern, often result in severe disfigurement, disability, or death; more than a third of deaths in the US are caused by head and neck injuries. With industrialization, developing nations are also recording increasing rates of traumatic accidents such as traffic accidents and gunshot wound [12].

As for South Korea, trauma is the third most common cause of death and has also long been discussed as a major social problem [6]. Amendment of laws on “emergency medical service act” in 2012 resulted in the government establishing Level I trauma centers (officially referred to as “Regional trauma centers” in South Korea) with the aim of reducing the preventable trauma death rate. Each province now has a regional trauma center, modelled upon the trauma centers of nations with advanced health systems such as the US, all within an hour’s reach of every patient [7].

Surgeons perform the focal role in the multidisciplinary endeavor of trauma care, of which trauma surgery, neurosurgery, and orthopedic surgery are known as core areas. However, while plastic surgeons have been historically indispensable in reconstruction of posttraumatic defects, their role in Level I trauma centers around the world has not yet been clearly approved [14]. This study aims to assess the contribution of plastic surgeons in major trauma care by evaluating the characteristics of trauma patients underwent plastic surgery at a Level I trauma center.

## Materials and methods

### 1. Study subjects

This retrospective study adheres to institutional guidelines and was approved by the Institutional Review Board of Dankook university hospital (IRB No. DKUH-2021-03-001). The study was designated to review data on trauma patients (with Injury Severity Score [ISS] over nine points) recorded from November 2014 to October 2020 in the trauma registry system of our hospital. More than 15 points of ISS are usually considered severe trauma patients, but more than 9 points are regarded as moderate one, so 9 points or more are sometimes targeted in recent trauma studies [15]. Meanwhile, the trauma registry system, which was monitored nationwide, contained all of the patient’s information, from their trauma history and initial vital signs to the location and severity of their injuries.

### 2. Propensity score matching (PSM)

Before attempting to determine the factors that affect plastic surgery participation, we performed propensity score matching to reduce the statistical bias from the differing sizes of the PS and Non-PS groups. For PSM, logistic regression was used to obtain the predicted probability. The matching variables for the two groups were age, sex, initial vital signs (blood pressure, heart rate, respiratory rate, and body temperature in the emergency room) with a caliper of 0.25.

### 3. Severity score

The severity of trauma injury is categorized into anatomical, physiological, and comorbidity systems. We referred to the Abbreviated Injury Scale (AIS) and Injury Severity Score (ISS) for anatomical measurements, and the Revised Trauma Score (RTS) for physiological measurements. In general, the AIS checks six predefined body regions (Head and Neck, Face, Chest, Abdomen, Extremity, and External), each ranging from 1 (minimal) to 6 (maximal). The ISS, another anatomical severity index, is calculated using the AIS, which is the sum of the squared top three AIS values. The ISS ranges from 1 to 75 points, and usually divides into groups 1–8 points (minor), 9–15 points (moderate), 16–24 points (serious), 25–49 points (severe), 50–74 points (critical), and 75 points (maximal) [7,16]. Meanwhile, the RTS is a physiological indicator that is highly related to mortality in trauma patients, and its score ranges from 0 to 7.84 points with lower scores representing a poor prognosis.

In this study, the ISS divided the subjects into three groups of 9–15, 16–24, and over 25 points. RTS also divided patients into three groups of 7.00–7.84, 6.00–6.99, and less than 6 points. Among the AIS subcategories, ‘Head and Neck (H&N)’ and ‘Face’ were investigated. These two subcategories were based on the results of our previous epidemiology study that the plastic surgery treated over 95% of trauma patients with facial injuries [7].

### 4. Treatment outcomes

Factors of treatment outcomes considered were the duration of hospitalization [total duration and stays in intensive care unit (ICU)], rates of ICU admission, the post-discharge progress (discharge to home, transfer to another hospital, leaving hospital against medical advice, death, and etc.).

### 5. Covariates

The institution’s trauma registry system served as a source for more information on all trauma patients, such as age, sex, cause of trauma, vital signs in the emergency room (body temperature, heart rate, systolic and diastolic blood pressure, and respiratory rate), and number of operations. The cause of trauma included traffic accidents, falls, collision, injuries from objects, suffocation, burns, and unknown causes. The number of operations was divided into three groups (0, 1, and over 2).

### 6. Statistical analysis

Chi-square test and ANOVA test were conducted to review the distribution of demographic characteristics between PS and Non-PS groups. Severity factors (ISS, RTS) were analyzed with chi-square, ANOVA, and logistic regression test to assess the correlation between the two groups. In AIS, the distribution of the H&N and facial injuries across the covariates were analyzed with the chi-square test. The treatment outcomes were evaluated with linear and logistic regression analysis after adjusting for severity factors. All regression results were presented with the corresponding crude odds ratios (ORs) with 95% confidence intervals (CIs). A *p* value of < 0.05 was considered statistically significant. Statistical analysis was performed using R software version 4.04 (R Foundation, Vienna, Austria).

## Results

**Fig 1:**
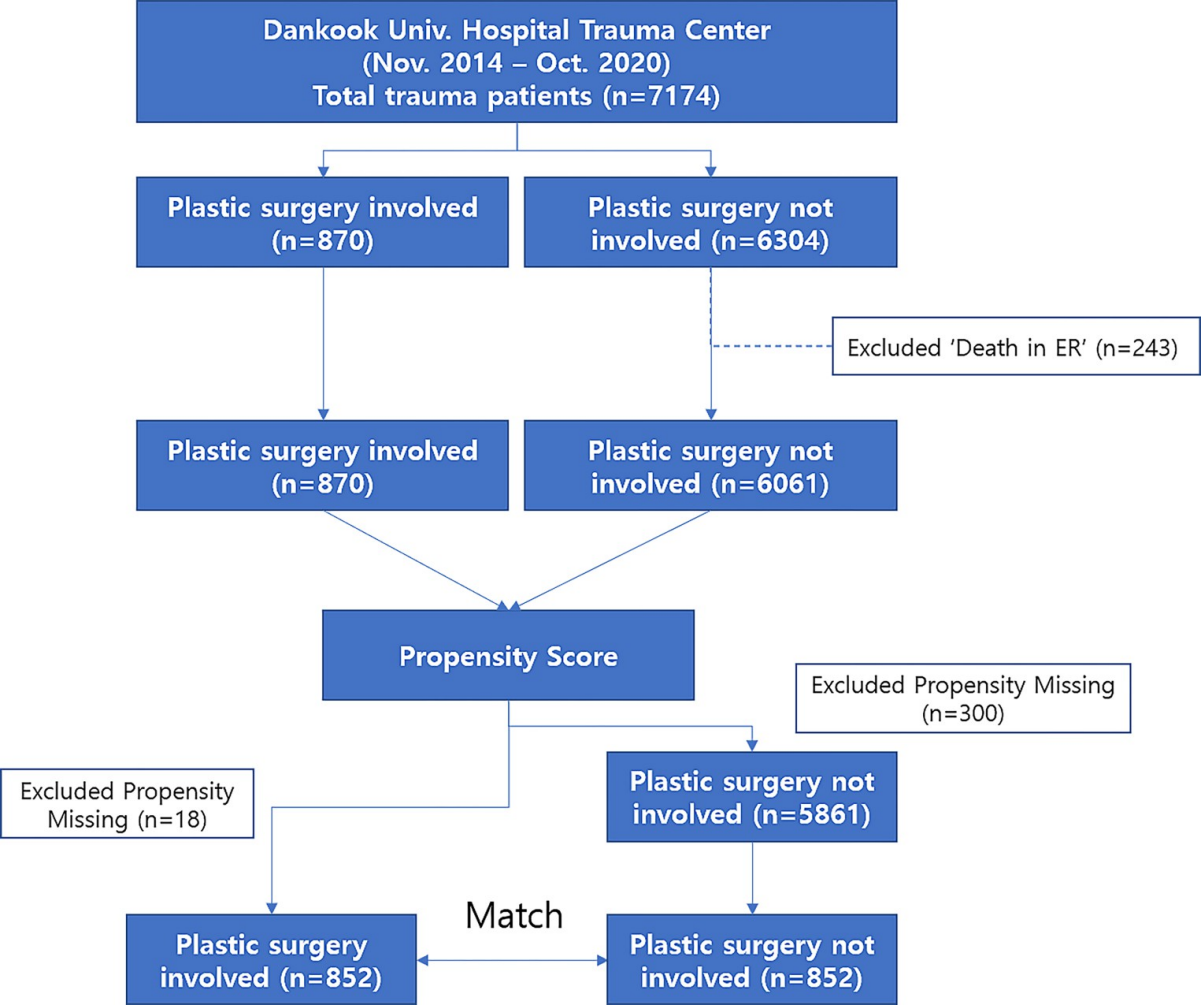
Algorithm of study sampling.

Of all of 7174 patients, the plastic surgery (PS) department treated 870 patients; the 6304 patients not treated by the PS were classified as the Non-PS. After deaths in the emergency room and patients with missing data were excluded from the study, 870 patients in the PS and 6061 patients in the Non-PS were finally enrolled. After PSM, the PS and Non-PS groups matched 1:1, with 852 patients per group, amounting to a total of 1704 subjects for this study (Fig 1).

### 1. Demographic analysis (PS vs. Non-PS group, Table 1)

The age, sex, and cause of trauma, which significantly differed between PS and Non-PS groups in analysis of all samples, were not significantly different after propensity score matching (PSM) (age, *p* < 0.0001 vs. *p* = 0.1628; sex, *p* < 0.0001 vs. *p* = 0.6322; cause of trauma, *p* < 0.0001 vs. *p* = 0.7194). In PSM data, the middle-aged group (40–59 years) showed the highest trauma prevalence (40.0% vs. 35.5%), and males had been injured about four times more than the females (percentage of male and female, 78.9% and 21.1% in PS group vs. 79.8% and 20.2% in Non-PS group). As for causes of trauma, traffic accidents were the most common cause in both groups (62.2% vs. 65.3%).

**Table 1:** Characteristics of trauma patients between two groups.

Variable	Total (n = 6931)			PSM (n = 1704)		
	PS (n = 870)	Non-PS (n = 6061)	*p* value	PS (n = 852)	Non-PS (n = 852)	*p* value
Age (years)			**< 0.0001**			< 0.1628
< 20	58 (6.7%)	289 (4.8%)		55 (6.5%)	67 (7.9%)	
20 ~ 39	178 (20.5%)	798 (13.2%)		178 (20.9%)	166 (19.5%)	
40 ~ 59	348 (40.0%)	1897 (31.3%)		341 (40.0%)	302 (35.5%)	
60 ~ 79	230 (26.4%)	2172 (35.8%)		222 (26.1%)	252 (29.6%)	
≥ 80	56 (6.4%)	905 (14.9%)		56 (6.6%)	65 (7.6%)	
Sex			**< 0.0001**			< 0.6322
Male	689 (79.2%)	4102 (67.7%)		672 (78.9%)	680 (79.8%)	
Female	181 (20.8%)	1959 (32.3%)		180 (21.1%)	172 (20.2%)	
Cause of trauma			**< 0.0001**			< 0.7194
Traffic accident	542 (62.3%)	2640 (43.6%)		530 (62.2%)	556 (65.3%)	
Fall down	156 (17.9%)	1223 (20.2%)		155 (18.2%)	149 (17.5%)	
Collision	109 (12.5%)	1770 (29.2%)		108 (12.7%)	095 (11.2%)	
Object	15 (1.7%)	276 (4.6%)		15 (1.8%)	17 (2.0%)	
Suffocation	0 (0.0%)	13 (0.0%)		0 (0.0%)	0 (0.0%)	
Burn	2 (0.0%)	4 (0.0%)		2 (0.2%)	1 (0.1%)	
Unknown	46 (5.3%)	135 (2.2%)		42 (4.9%)	34 (4.0%)	
ISS			**< 0.0001**			**< 0.0001**
9 ~ 15	478 (54.9%)	3528 (58.2%)		474 (55.6%)	472 (55.4%)	
16 ~ 24	281 (32.3%)	1369 (22.6%)		276 (32.4%)	214 (25.1%)	
≥ 25	111 (12.8%)	1164 (19.2%)		102 (12.0%)	166 (19.5%)	
Mean ± SD	16.44 ± 7.15	16.01 ± 8.90	< 0.1720	16.29 ± 7.04	16.68 ± 9.16	< 0.3221
RTS			**< 0.0459**			**< 0.0316**
≥ 7.00	742 (85.3%)	4914 (81.1%)		739 (88.5%)	706 (85.5%)	
6.00 ~ 6.99	58 (6.7%)	415 (6.8%)		58 (7.0%)	57 (6.9%)	
< 6.00	38 (4.4%)	385 (6.4%)		38 (4.6%)	63 (7.6%)	
Uncheck	32 (3.7%)	347 (5.7%)		17 (2.0%)	26 (3.1%)	
Mean ± SD	7.64 ± 0.58	7.56 ± 0.77	**< 0.0033**	7.64 ± 0.58	7.55 ± 0.78	**< 0.0044**
AIS (H&N)			**< 0.0001**			**< 0.0001**
Yes	568 (65.3%)	2676 (44.2%)		554 (65.0%)	395 (46.4%)	
No	302 (34.7%)	3385 (55.8%)		298 (35.0%)	457 (53.6%)	
Odd Ratio	2.379	Ref	**< 0.0001**	2.151	Ref	**< 0.0001**
AIS (Face)			**< 0.0001**			**< 0.0001**
Yes	769 (88.4%)	1150 (19.0%)		755 (88.6%)	224 (26.3%)	
No	101 (11.6%)	4911 (81.0%)		097 (11.4%)	628 (73.7%)	
Odd Ratio	032.514	Ref	**< 0.0001**	021.822	Ref	**< 0.0001**
Frequency of operation (n)			**< 0.0001**			**< 0.0001**
0	332 (38.2%)	2206 (36.4%)		325 (38.2%)	324 (38.0%)	
1	269 (30.9%)	2568 (42.4%)		261 (30.6%)	331 (38.9%)	
≥ 2	269 (30.9%)	1287 (21.2%)		266 (31.2%)	197 (23.1%)	
Discharge			**< 0.0001**			**< 0.0001**
Home	568 (65.3%)	3211 (53.0%)		559 (65.6%)	471 (55.3%)	
Transfer	204 (23.4%)	2028 (33.5%)		200 (23.5%)	272 (31.9%)	
AMA	10 (1.1%)	81 (1.3%)		10 (1.2%)	11 (1.3%)	
Death	12 (1.4%)	486 (8.0%)		010 (1.2%)	55 (6.5%)	
Etc.^a^	76 (8.7%)	255 (4.2%)		73 (8.6%)	43 (5.1%)	

n, numbers; Data are presented as n (%).

PSM, propensity score matching; PS, department of plastic surgery; H&N, head and neck; SD, standard deviation; ISS, Injury Severity Score; RTS, Revised Trauma Scale; AIS, Abbreviated Injury Score; AMA, against medical advice.

^a^ It included unrecorded (due to no admission in our hospital) and unidentified cases.

The mean ISS showed no significant difference between the two groups in PSM sample (16.29 ± 7.04 points vs. 16.68 ± 9.16 points, *p* = 0.3221). Meanwhile, the mean RTS showed a significant difference between the two groups in PSM sample (7.64 ± 0.58 vs. 7.55 ± 0.78, *p* = 0.0044). According to the AIS in PSM sample, both H&N (65.0%) and face (46.4%) categories were significantly more common in the PS group (*p* < 0.0001), and their contribution ratios were 2.151 and 21.822 times, respectively.

During hospitalization, there were significantly more operations in the PS group (31.2% vs. 23.1%, *p* < 0.0001). The ratio of patients who went home on discharge was also significantly higher in the PS group (65.6% vs. 55.3%, *p* < 0.0001).

### 2. Injury Severity Score analysis (PS vs. Non-PS group in PSM sample, Table 2)

In the logistic regression analysis of ISS, the odds ratio was 0.998 when the Non-PS group was a reference (95% CI 0.985–1.010, *p* = 0.7051). The PS group’s mean value of categorized ISS subgroups was significantly higher in the moderate (9–15) and serious (16–24) subgroups (11.52 ± 2.13 points vs. 10.56 ± 1.93 points, *p* < 0.0001 in moderate subgroup; 19.15 ± 2.48 points vs. 18.62 ± 2.48 points, *p* = 0.0199), but the difference was not proven in the severe (over 25) subgroup (30.72 ± 6.29 points vs. 31.57 ± 8.92 points, *p* = 0.3968).

**Table 2:** Regression analysis of Injury Severity Score (ISS).

ISS	Group	Total						PSM					
		N	Mean ± SD	*p* value	OR	95% CI	*p* value	N	Mean ± SD	*p* value	OR	95% CI	*p* value
Total	PS	870	16.44 ± 7.15	< 0.1720	1.009	1.000 ~ 1.017	**< 0.0432**	852	16.29 ± 7.04	< 0.3221	0.998	0.985 ~ 1.010	< 0.7051
	Non-PS	6061	16.01 ± 8.90		1	Ref		852	16.68 ± 9.16		1	Ref	
09 ~ 15	PS	478	11.52 ± 2.13	**< 0.0001**	1.412	1.349 ~ 1.479	**< 0.0001**	474	11.52 ± 2.13	**< 0.0001**	1.253	1.176 ~ 1.335	**< 0.0001**
	Non-PS	3528	10.12 ± 1.76		1	Ref		472	10.56 ± 1.93		1	Ref	
16 ~ 24	PS	281	19.20 ± 2.50	**< 0.0001**	1.114	1.056 ~ 1.174	**< 0.0001**	276	19.15 ± 2.48	**< 0.0199**	1.099	1.020 ~ 1.183	**< 0.0126**
	Non-PS	1369	18.55 ± 2.36		1	Ref		214	18.62 ± 2.48		1	Ref	
≥ 25	PS	111	30.65 ± 6.26	< 0.7670	0.994	0.966 ~ 1.022	< 0.6505	102	30.72 ± 6.29	< 0.3968	0.989	0.954 ~ 1.024	< 0.5271
	Non-PS	1164	30.88 ± 7.89		1	Ref		166	31.57 ± 8.92		1	Ref	

N, numbers; SD, standard deviation; OR, odd ratio; CI, confidence interval; PSM, propensity score matching; PS, department of plastic surgery.

### 3. Abbreviated Injury Scale (head and neck, face) analysis (H&N vs. face in PSM sample, Table 3)

Of all the trauma patients, H&N and face injuries accounted for 46.8% and 27.7%, respectively. H&N injury was more common in fall trauma (62.2%) than in traffic accidents (54.8%). In correlation analysis with the ISS, the percentage of H&N injured patients was significantly higher than face injured patients in the severe subgroup (79.9% vs. 54.5%, *p* < 0.0001). The RTS reflecting with the Glasgow Coma Scale also significantly decreased in H&N injured patients (91.1% vs. 56.4% in RTS < 6.00 subgroup, *p* < 0.0001). Meanwhile, there were significantly more face injury patients (61.8%) than H&N patients (55.0%) who were sent home on discharge (*p* < 0.0001).

**Table 3:** Analysis of Abbreviated Injury Scale (Head and neck, Face).

Variable	Total							PSM						
	N	H&N	%	*p* value	Face	%	*p* value	N	H&N	%	*p* value	Face	%	*p* value
Total	6931	3244	46.8%		1919	27.7%		1704	949	55.7%		979	57.5%	
Age				**< 0.0001**			**< 0.0001**				**< 0.0091**			**<** 0.0901
< 20	0347	0179	51.6%		0118	34.0%		0122	077	63.1%		073	59.8%	
20 ~ 39	0976	0434	44.5%		0364	37.3%		0344	167	48.6%		202	58.7%	
40 ~ 59	2245	1062	47.3%		0697	31.0%		0643	356	55.4%		382	59.4%	
60 ~ 79	2402	1256	52.3%		0597	24.9%		0474	284	60.0%		266	56.1%	
≥ 80	0961	0313	32.6%		0143	14.9%		0121	065	53.7%		056	46.3%	
Sex				**< 0.0001**			**< 0.0001**				**< 0.0113**			**<** 0.2637
Male	4791	2439	50.9%		1504	31.4%		1352	774	57.3%		786	58.1%	
Female	2140	0805	37.6%		0415	19.4%		0352	175	49.7%		193	54.8%	
Cause of trauma				**< 0.0001**			**< 0.0001**				**< 0.0001**			**< 0.0001**
Traffic accident	3182	1645	51.7%		1254	39.4%		1086	595	54.8%		668	61.5%	
Fall down	1379	0744	54.0%		0366	26.5%		0304	189	62.2%		180	59.2%	
Collision	1879	0702	37.4%		0233	12.4%		0203	127	62.6%		103	50.7%	
Object	0291	0065	22.3%		0038	13.1%		0032	014	43.8%		013	40.6%	
Suffocation	0013	0009	69.2%		0000	00.0%		0000	000	00.0%		000	00.0%	
Burn	0006	0001	16.7%		0001	16.7%		0003	001	33.3%		001	33.3%	
Unknown	0181	0078	43.1%		0027	14.9%		0076	023	30.3%		014	18.4%	
ISS				**< 0.0001**			**< 0.0001**				**< 0.0001**			**< 0.0084**
9 ~ 15	4006	1225	30.6%		0890	22.2%		0946	409	43.2%		523	55.3%	
16 ~ 24	1650	1012	61.3%		0607	36.8%		0490	326	66.5%		310	63.3%	
≥ 25	1275	1007	79.0%		0422	33.1%		0268	214	79.9%		146	54.5%	
RTS				**< 0.0001**			**< 0.0001**				**< 0.0001**			**<** 0.2611
≥ 7.00	5656	2208	39.0%		1465	25.9%		1445	729	50.5%		820	56.8%	
6.00 ~ 6.99	0473	0360	76.1%		0176	37.2%		0115	090	78.3%		076	66.1%	
< 6.00	0423	0376	88.9%		0151	35.7%		0101	092	91.1%		057	56.4%	
Uncheck	0379	0300	79.2%		0127	33.5%		0043	038	88.4%		026	60.5%	
Frequency of operation (n)				**< 0.0001**			**< 0.0001**				**< 0.0004**			**< 0.0001**
0	2538	1533	60.4%		0704	27.7%		0649	397	61.2%		343	52.9%	
1	2837	0960	33.8%		0632	22.3%		0592	296	50.0%		328	55.4%	
≥ 2	1556	0751	48.3%		0583	37.5%		0463	256	55.3%		308	66.5%	
Discharge				**< 0.0001**			**< 0.0222**				**< 0.0001**			**< 0.0001**
Home	3779	1630	43.1%		1107	29.3%		1030	566	55.0%		637	61.8%	
Transfer	2232	1093	49.0%		0582	26.1%		0472	277	58.7%		254	53.8%	
AMA	0091	0045	49.5%		0023	25.3%		0021	015	71.4%		012	57.1%	
Death	0498	0354	71.1%		0128	25.7%		0065	050	76.9%		029	44.6%	
Etc.^a^	0331	0122	36.9%		0079	23.9%		0116	041	35.3%		047	40.5%	

N, numbers; PSM, propensity score matching; H&N, head and neck; ISS, Injury Severity Score; RTS, Revised Trauma Scale; AMA, against medical advice.

^a^ It included unrecorded (due to no admission in our hospital) and unidentified cases.

### 4. Revised Trauma Score analysis (PS vs. Non-PS group in PSM sample, Table 4)

A comparison of the mean RTS of all patients in the PSM sample revealed a significant difference between the PS and Non-PS groups (7.64 ± 0.58 points vs. 7.55 ± 0.78 points, *p* = 0.0044). However, comparing the mean RTS of the PS and Non-PS groups within each of the three subgroups of 7.00–7.84, 6.00–6.99, and less than 6 points RTS, did not reveal a significant difference in any subgroup.

**Table 4:** Regression analysis of Revised Trauma Score (RTS).

RTS	Group	Total						PSM					
		N	Mean ± SD	*p* value	OR	95% CI	*p* value	N	Mean ± SD	*p* value	OR	95% CI	*p* value
Total	PS	838	7.64 ± 0.58	**0.0033**	1.191	1.059 ~ 1.339	**0.0035**	835	7.64 ± 0.58	**0.0044**	1.230	1.065 ~ 1.422	**0.0050**
	Non-PS	5715	7.56 ± 0.77		1	Ref		826	7.55 ± 0.78		1	Ref	
≥ 7.00	PS	742	7.82 ± 0.11	0.2591	1.521	0.732 ~ 3.158	0.2609	739	7.82 ± 0.11	0.2307	1.739	0.700 ~ 4.319	0.2331
	Non-PS	4914	7.82 ± 0.12		1	Ref		706	7.82 ± 0.12		1	Ref	
6.00 ~ 6.99	PS	58	6.81 ± 0.22	0.3803	1.827	0.474 ~ 7.048	0.3814	58	6.81 ± 0.22	0.9183	1.095	0.198 ~ 6.058	0.9174
	Non-PS	415	6.78 ± 0.22		1	Ref		57	6.81 ± 0.22		1	Ref	
< 6.00	PS	38	5.40 ± 0.71	0.1499	1.345	0.896 ~ 2.019	0.1522	38	5.40 ± 0.71	0.2554	1.319	0.819 ~ 2.123	0.2549
	Non-PS	385	5.16 ± 1.02					63	5.19 ± 0.99		1	Ref	

N, numbers; SD, standard deviation; OR, odd ratio; CI, confidence interval; PSM, propensity score matching; PS, department of plastic surgery.

### 5. Treatment outcomes analysis (PSM sample, Table 5)

The length of stay (LOS) of total admission was 22.13 ± 26.56 days in the PS group and 25.06 ± 31.86 days in the Non-PS group (adjusted variable; ISS, *p* = 0.0585 and RTS, *p* = 0.0680). From the PS group, 91.7% went to the ICU; the odds ratio of the PS and Non-PS groups admitting to the ICU was 0.555 when ISS was used as the adjustment factor (95% CI 0.373–0.826, *p* = 0.0037). The ICU LOS was 3.21 ± 8.69 days in the PS group and 4.36 ± 11.59 days in the Non-PS group (adjusted variable; ISS, *p* = 0.0211 and RTS, *p* = 0.1753). The value of beta (*β*) was– 1.15 after linear regression analysis with ISS as the adjustment factor. The odds ratios of whether the PS vs. the Non-PS groups were discharged to go home were 1.550 (95% CI 1.266–1.896, *p* < 0.0001) when adjusted with ISS, and 1.451 (95% CI 1.185–1.776, *p* < 0.0001) when adjusted with RTS.

**Table 5:** Regression analysis of treatment outcomes.

Group			Total		PSM	
			PS	Non-PS	PS	Non-PS
Numbers			870	6061	852	852
Total LOS	Mean ± SD (d)		22.21 ± 26.58	23.75 ± 27.65	22.13 ± 26.56	25.06 ± 31.86
	ISS	ß	−1.77	Ref	−2.64	Ref
		SE	−1.00		−1.39	
		*p* value	−0.0716		−0.0585	
	RTS	ß	−2.07	Ref	−2.48	Ref
		SE	−0.97		−1.36	
		*p* value	**−0.0318**		−0.0680	
ICU LOS	Mean ± SD (d)		3.28 ± 8.69	4.07 ± 10.65	3.21 ± 8.69	4.36 ± 11.59
	ISS	ß	−0.96	Ref	−1.15	Ref
		SE	−0.36		−0.50	
		*p* value	**−0.0070**		**−0.0211**	
	RTS	ß	−0.46	Ref	−0.57	Ref
		SE	−0.32		−0.42	
		*p* value	−0.1448		−0.1753	
ICU Admission	Yes (n)		796 (91.5%)	5815 (96.0%)	781 (91.7%)	811 (95.2%)
	ISS	OR	0.443	Ref	0.555	Ref
		95% CI	0.337 ~ 0.580		0.373 ~ 0.826	
		*p* value	**< 0.0001**		**0.0037**	
	RTS	OR	0.470	Ref	0.561	Ref
		95% CI	0.355 ~ 0.621		0.374 ~ 0.842	
		*p* value	**< 0.0001**		**0.0053**	
Final outcome	Home (n)		568 (65.3%)	3211 (53.0%)	559 (65.6%)	471 (55.3%)
	ISS	OR	1.767	Ref	1.550	Ref
		95% CI	1.518 ~ 2.058		1.266 ~ 1.896	
		*p* value	**< 0.0001**		**< 0.0001**	
	RTS	OR	1.561	Ref	1.451	Ref
		95% CI	1.338 ~ 1.822		1.185 ~ 1.776	
		*p* value	**< 0.0001**		**< 0.0001**	

d, days; n, numbers.

PSM, propensity score matching; PS, department of plastic surgery; LOS, length of stay; SD, standard deviation; ICU; intensive care unit; ISS, Injury Severity Score; RTS, Revised Trauma Scale; SE, standard error; OR, odd ratio; CI, confidence interval.

### 6. Analysis between AIS and prognosis (H&N vs. Face, Table 6)

While patients in the H&N category ranged from 1 to 6 in AIS scores, those in the face category had AIS scores of no more than five. The significance was not reviewed because there were duplicate patients in both groups. Instead, we identified only the tendency between the two AIS categories and the treatment outcomes. Within the same AIS scores, patients in the face category showed poor overall outcomes (RTS, total and ICU LOS). This tendency was particularly marked in patients with an AIS score of 3. Compared to H&N patients, face category patients had approximately 2–4 times longer total and ICU LOS (20.55 ± 24.68 days vs. 41.75 ± 50.23 days in total LOS; 4.53 ± 10.11 days vs. 21.33 ± 27.36 days in ICU LOS).

**Table 6:** Analysis between AIS and prognosis.

AIS score	Category	N	RTS	Total LOS	ICU LOS	ICU Admission rate (%)
1 (Minor)	H&N	0188	7.59 ± 0.67	20.76 ± 20.28	01.66 ± 3.83	95.74%
	Face	1071	7.55 ± 0.73	22.30 ± 25.57	04.34 ± 9.66	95.52%
2 (Moderate)	H&N	0432	7.53 ± 0.79	24.55 ± 27.25	04.26 ± 9.73	95.60%
	Face	0821	7.48 ± 0.81	25.17 ± 27.86	04.87 ± 13.47	96.47%
3 (Serious)	H&N	1352	7.59 ± 0.64	20.55 ± 24.68	04.53 ± 10.11	96.15%
	Face	0024	6.07 ± 1.57	41.75 ± 50.23	21.33 ± 27.36	100%
4 (Severe)	H&N	0559	7.41 ± 0.90	30.31 ± 30.54	08.16 ± 12.5	98.57%
	Face	0003	4.62 ± 0.17	24.00 ± 38.12	23.33 ± 36.96	100%
5 (Critical)	H&N	0707	6.61 ± 1.34	31.40 ± 42.10	13.04 ± 20.00	95.76%
	Face	0000	-	-	-	-
6 (Maximal)	H&N	0006	5.72 ± 2.03	25.17 ± 15.38	24.17 ± 13.82	100%
	Face	0000	-	-	-	-

AIS, Abbreviated Injury Score; H&N, head and neck; N, numbers, RTS, revised trauma scale; LOS, length of stay; ICU, intensive care unit.

## Discussion

Traumatic and burn injuries are a principal cause of morbidity and mortality. In the United States, regardless of socioeconomic background and race, they are the most common cause of death between 1 and 44 years of age, with hundreds of thousands of cases admitted to hospitals each year [10]. Worldwide, trauma was the cause of 5.8 million deaths in 2012, and the number is expected to post a steady increase [7]. With the International Red Cross’ approximations of 80 million people being impacted by such occurrences each year, the economic, social and psychological impact is substantial [17]. Such is the need for organized efforts to address the burden of traumatic events. In the US, the initial guidance on trauma care set forth by the American College of Surgeons in 1976 served as the foundation for the Level I trauma centers of today [7,13,18]. The following years, until the early 2000s, saw “trauma systems” built in every US state [18]. Such developments have contributed to better patient outcomes and have also provided a model for other countries’ trauma response systems [7,13,19].

According to the 2008 annual report from the Korea Disease Control and Prevention Agency, trauma was the third major cause of death after cancer and cerebrovascular disease. Moreover, 35% of preventable trauma death rate from trauma in South Korea were found, recording more than twice of that of other advanced economies. Amendment of laws regulating “emergency medical service act” in 2012 provided the foundation for the government to build Level I trauma centers (named as “regional trauma centers” in South Korea) modelled on schemes from the US. Since the first five regional trauma centers were operated in November 2014, each province now has its own trauma center, providing access for all patients within an hour. The centers also act as a control tower for transportation and care of trauma patients as well as enhancing the regional health care system [6,7].

The nation designed trauma centers are funded by the government, including payment for full-time, “dedicated” specialists and on-call compensation. Currently only seven specialties are eligible for the position of the dedicated specialist: emergency medicine, cardiothoracic surgery, general surgery, orthopedic surgery, neurosurgery, radiology, and anesthesiology [6,7]. At the same time, the regional trauma center maintains a roster of on-call specialists from internal medicine, ophthalmology, urology, obstetrics and gynecology, pediatrics, otorhinolaryngology, dentistry, and plastic surgery. These specialists only serve as “supporting specialists”, whose work in the trauma center is not government-funded [6,7]. The current imbalance of support in the form of government funding to the multidisciplinary approach remains a subject of criticism. Indeed, plastic surgeons are equipped with the skill sets required to address traumatic injuries, including management of soft-tissue injury, fractures in facial structures, neurovascular injury, and salvaging extremities [5–8]. Such critical role of plastic surgeons in trauma care is underrecognized due to regulations preventing them from serving as dedicated specialists, thereby depriving them of commensurate compensation. Even in developed trauma care systems such as the United States and the United Kingdom, this tendency is also prevalent [14,20].

Trauma centers worldwide often provide care to patients with maxillofacial injuries. According to the Major Trauma Outcome Study report in 1995, 34% of patients with trauma had a mid-face injury and 25% had facial bone fractures [21]. The severity of facial injuries varies widely, from minor chin lacerations and chipped teeth to life-threatening injuries across the panfacial area that require complex airway management and extended intensive care, including surgery [11,22,23]. In children, facial fractures may cause enduring damage. Indeed, many victims of pediatric maxillofacial trauma experience deformities and functional impairments. In the US, facial injuries cost $1.2 billion in healthcare expenses annually [19,24]. The complexities of craniofacial injuries often require specialized care from plastic surgeons, as demonstrated by the fact that nearly 40% of such injuries are treated with plastic surgery, as opposed to the 0.5% treated by general surgeons [12]. In today’s increasingly diverse medical system, treating trauma patients with multiple injuries requires a multidisciplinary approach. Multidisciplinary care is vital in the treatment of trauma patients whose golden time is critical. Even if it is possible to manage facial damage in general surgery department, it is far effective to treat patients concurrently with plastic surgery, which has more experience in facial surgery. In our study, we found a strong relationship between the face injury and plastic surgery, with contribution ratio was 21.822 times.

Meanwhile, the severity of trauma patients could be evaluated in various viewpoints. In general, severity of trauma injuries is assessed across anatomical, physiological, and comorbidity systems. The Injury Severity Score (ISS) and the Abbreviated Injury Scale (AIS) are used as anatomical severity indicators, while the Glasgow Coma Scale (GCS) and the Revised Trauma Score (RTS) are physiological indices. The Trauma and Injury Severity Score (TRISS) and Severity Characterization of Trauma (ASCOT) reflect both anatomical and physiological properties [16,25]. Among them, we analyzed the trauma severity in patients using ISS, AIS, and RTS. While severe trauma patients are usually defined as patients with over 15 points ISS, this definition is arbitrary. The current study included patients with over 9 ISS. A several literatures have employed ISS groups that define patients with ISS greater than 9 as severe trauma patients [15,26].

Because plastic surgery is not typically viewed as a life-saving procedure, most trauma surgeons tend to underestimate the severity of trauma patients who have been treated by plastic surgeons. However, our results contradicted this perception; there was no significant difference in the mean ISS between the PS and Non-PS in both the entire group of subjects and PSM samples. This demonstrates that the severity index of trauma patients is less correlated with whether they were treated in plastic surgery or not. These results were somewhat different from the results of a British plastic surgeon’s workload study conducted by Hendrickson SA et al. [20]. Also, bearing in mind that AIS is a categorical value with variable criteria [15,25], we compared AIS scores and treatment outcomes of patient in the H&N and face category. The facial injury patients showed poorer outcomes compared to H&N patients with the same AIS scores of 3 points, with approximately twice as longer stays in the hospital, and four times longer stays in the ICU. We therefore believe the AIS scoring criteria in the face category underestimates the severity of the injuries, and suggest the criteria be revised.

Unlike the ISS, the RTS was significantly higher in the plastic surgery group in both the entire sample and PSM samples. The correlation between ISS and RTS in regression analysis was moderate (−0.4628, *p* < 0.001). It is worth noting that while the ISS is evaluated afterwards, the RTS is determined at the initial patient workup, when vital signs are first taken. The difference in the RTS between PS and the Non-PS groups might be attributed to the fact that plastic surgeons are typically notified of a trauma patient only after their vital signs have stabilized. As stable vital signs mean higher RTS scores, the plastic surgeon is more likely to treat patients with high RTS scores, possibly resulting in high RTS for the PS group in this study.

The current study presents data to quantify the contribution of plastic surgeons in major trauma care by evaluating the characteristics of trauma patients underwent plastic surgery at a Level I trauma center. The data herein will hopefully contribute to better trauma response strategies and future human resource plans. Nonetheless, this study has several limitations. First, because this retrospective study was carried out at a single trauma center, it is not representative of South Korean regional trauma centers in general. Second, given the nature of plastic surgery which is typically performed on patients with relatively stable vital signs, we were compelled to exclude those who died in the emergency room. Third, we were not able to consider other factors that may have affected patients’ outcomes such as previous medical history and family history, because in the initial evaluation, it is difficult to obtain such personal information on severe trauma patients. Lastly, depending on the hospital, the triage of clinical tasks may vary. For instance, at our hospital, hand trauma has been triaged by the plastic surgery and orthopedic surgery, while mandibular fractures have been triaged by the plastic surgery and dental surgery.

## Conclusion

This article underscores the specialty of plastic surgery and its substantial role in the multidisciplinary work of regional trauma centers. The characteristics of trauma patients who received plastic surgery treatment are also identified in the severity analysis. We thus argue that plastic surgical care is imperative for trauma patients, and expect to be implicated in trauma system planning.
